# Associations between polymorphisms in *ELOVL2* and *OSBPL8* genes and feed efficiency in meat-type ducks

**DOI:** 10.1016/j.psj.2026.107118

**Published:** 2026-05-13

**Authors:** Lianzhen Lu, Shuang Gu, Guiru Qiu, Taikang Zhang, Zihao Tang, Haoming Chang, Jiafa Wang, Zhaoyu Geng, Sihua Jin

**Affiliations:** a College of Animal Science and Technology, Anhui Agricultural University, Hefei 230036, China; b School of Public Health, Anhui Medical University, Hefei 230032, China; c Huangshan Qiangying Duck Breeding, Co. Ltd., Huangshan 245461, China

**Keywords:** Duck, Feed efficiency, *ELOVL2*, *OSBPL8*, Association analysis

## Abstract

Feed efficiency (**FE**) is important in the poultry industry because feed accounts for 70% of total poultry production expenses. The elongation of very-long-chain fatty acid protein 2 (***ELOVL2***) and oxysterol binding protein-like 8 (***OSBPL8***) genes are promising candidates for influencing FE owing to their potential roles in lipid metabolism. The genetic effects by which *ELOVL2* and *OSBPL8* influence FE remain unclear. Therefore, this study aimed to investigate the associations between SNPs in *OSBPL8* and *ELOVL2* and FE traits in meat-type ducks. Genomic DNA was extracted from 505 ducks for primer design, PCR amplification, and target gene sequencing. Polymorphism analysis identified two SNPs in *OSBPL8* (g.40194710T > A and g.40194759A > G) and two in *ELOVL2* (g.95803706 G > A and g.95803927 G > A). Both *ELOVL2* SNPs were significantly associated with feed conversion ratio (**FCR**), residual feed intake (**RFI**) (*P* < 0.01), and ADFI (*P* < 0.05). For *OSBPL8*, the g.40194710T > A locus was associated with FCR and ADFI (*P* < 0.05), whereas the g.40194759A > G locus was associated with RFI (*P* < 0.05). Notably, combined genotype analysis indicated that the AAAATAAG genotype exhibited lower FCR and RFI values than the other genotype combinations (*P* < 0.05). In conclusion, polymorphisms in *OSBPL8* and *ELOVL2* are significantly associated with FE traits in meat-type ducks, offering valuable molecular markers for selecting breeding pairs with enhanced feed utilization ability.

## Introduction

Feed accounts for 70% of the total poultry production expenses, and is closely associated with feed efficiency (**FE**) (Aggrey et al., 2010; Guo et al., 2022). FE is an indicator of the relationship between feed intake (**FI**) and growth performance, and is commonly quantified using metrics such as residual feed intake (**RFI**) and feed conversion ratio (**FCR**). FCR is the amount of feed consumed per unit of product, such as meat, eggs, milk (Horodyska et al., 2017; Zhou et al., 2023). RFI is the difference between the actual and predicted FI of an animal based on its body weight and growth rate. RFI is a moderately heritable trait (Buddle et al., 2011), that is widely considered to be a more accurate and biologically independent indicator of FE than FCR in livestock and poultry (Cai et al., 2024; Ye et al., 2025a). High RFI values indicate a relatively low FE.

The elongation of very-long-chain fatty acids protein 2 (***ELOVL2***) gene encodes a key enzyme involved in the elongation of very-long-chain fatty acids (Obeid et al., 2023) and plays a central role in lipid metabolism (Nwagbo and Bernstein, 2023). Previous studies linked *ELOVL2* to FE-related traits across livestock species. In Hu sheep, *ELOVL2* was associated with energy metabolism in animals showing divergent RFI (Zhang et al., 2021). In poultry, *ELOVL2* regulates polyunsaturated fatty acid synthesis (Liu et al., 2023) and may carry an intronic SNP (rs313164887) associated with RFI in chicken (Wen et al., 2021). Integrated analyses of ducks identified *ELOVL2* as a candidate gene involved in lipid metabolism pathways under restricted feeding conditions, highlighting its pivotal role in adipose tissue deposition and energy utilization (Zhang et al., 2023). These findings collectively support the potential of *ELOVL2* as a key functional gene that could influence FE in ducks. Oxysterol binding protein-like 8 (***OSBPL8***), a member of the oxysterol binding protein family, participates in lipid metabolic regulation, intracellular transport, and homeostatic maintenance (Iung et al., 2018; Pietrangelo and Ridgway, 2018; Santos et al., 2020; Yan et al., 2008). *OSBPL8* is associated with milk fat content in Chinese Holstein cows (Li et al., 2014), underscoring its relevance for metabolic efficiency. However, the roles of *ELOVL2* and *OSBPL8* in ducks have been insufficiently investigated.

Owing to the need to reduce feed costs and the lack of validated molecular markers for FE in meat-type ducks, a candidate gene association study was conducted using a population of 505 meat-type ducks targeting *ELOVL2* and *OSBPL8*. SNPs were identified using PCR amplification and Sanger sequencing, and their associations with FCR and RFI were evaluated. Important genetic variants of *ELOVL2* and *OSBPL8* linked to FE in meat-type ducks were identified. The study offers molecular tools for marker-assisted breeder selection programs aimed at improving production sustainability and economic returns.

## Materials and methods

### Ethics statement

All animal care and experimental protocol in the present study were followed the guidelines of the Animal Care and Use Committee of Anhui Agricultural University in Hefei, China (permit number: SYXK 2016-007).

### Experimental animals and phenotypic measurements

Experimental meat-type ducks with complete pedigree information were provided by Huangshan Qiangying Duck Breeding Co., Ltd. (Anhui, China). All ducks were taken from genetically unrelated sources and maintained as closed populations for 13 generations based on their growth, FE, and feather color within each generation. They were weighed, sexed, wing-banded on the day of hatching, and raised on the floor for the first 2 weeks. At 21 d of age, 520 male ducks with similar body weights were transferred to individual cages to precisely measure FI. Feeding trials were conducted from 21 to 42 d of age. All ducks were fed the same diet *ad libitum* and reared under standardized conditions in accordance with the company’s protocols. The ingredients and nutrient levels of the basal diet are described in our previous study (Jin et al., 2025). Ducks that died during the trial or had incomplete phenotypic records were excluded, resulting in 505 experimental individuals included in the subsequent genetic and statistical analyses.

Body weights were recorded at 21 and 42 d of age, and the average daily gain (**ADG**) was calculated accordingly. Total feed intake for each duck during the 21–42 d period was recorded, and average daily feed intake (**ADFI**) was determined by dividing the total FI by the number of trial days. Based on the FI obtained from the ADG and ADFI, the FCR and RFI of each experimental duck were computed using an established formula (Dadfar et al., 2023). At 42 d of age, 3.5 mL of whole blood was collected from the wing vein of each duck into EDTA-coated tubes and stored at –20°C until DNA extraction.

### DNA extraction and primer design

Genomic DNA was extracted from the whole blood using the TIANamp Genomic DNA Kit (Tiangen Biochemical Technology Co., Ltd., Beijing, China). The procedure was performed as follows: 5 μL of whole blood was aliquoted, and lysis buffer GA was added to a total volume of 200 μL, followed by the addition of 25 μL Proteinase K and mixing. Afterward, 200 μL of buffer GB was added, and the mixture was vortexed thoroughly, briefly centrifuged, and incubated at 70 °C for 10 min until it became clear. After adding 200 μL of absolute ethanol and mixing for 15 s, the entire mixture was transferred to the adsorption column CB3 and centrifuged at 12, 000 rpm for 30 s. The column was then subsequently washed with 500 μL of buffer GD and 600 μL of wash buffer PW, each followed by centrifugation at 12, 000 rpm for 30 s; the washing with buffer PW was repeated once. The empty column was centrifuged at 12, 000 rpm for 2 min to remove residual ethanol, air-dried at ambient temperature with the cap open for 5 min, and then transferred to a new sterile tube. DNA was eluted by adding 100 μL of TE buffer to the center of the membrane, incubating for 5 min at ambient temperature, and centrifuging at 12, 000 rpm for 30 s. The eluted DNA was collected and stored at –20 °C. DNA quality was assessed by measuring the concentration and OD_260_/OD_280_ ratio using a Nanodrop 2000 spectrophotometer (Thermo Fisher Scientific, Waltham, MA, USA), and integrity was verified by 1.5% agarose gel electrophoresis at 120 V for 30 min. Primers for *OSBPL8* and *ELOVL2* were designed using Primer Premier 5.0 (PREMIER Biosoft International, Palo Alto, CA, USA) based on GenBank reference sequences. The primer sequences for *OSBPL8* and *ELOVL2* are listed in Table 1.

**Table 1:** Primer sequences used for PCR amplification of *ELOVL2* and *OSBPL8* in meat-type ducks.

Genes^1^	GenBank Accession no.	Chromosome	Sequences^2^ (5′ to 3′)	Tm/°C	Product Size/bp
*ELOVL2*	NC_051773.1	2	F:GGAACCCACCAGGAAAGAC	64.8	623
			R:GTGAGCCAAGGAGACCAAG		
*OSBPL8*	NC_051772.1	1	F:GAGTGCTGGAATGCTTATAC	58.0	647
			R:CCTAATCCTGGCTTCTAAT		

1 *ELOVL2*: elongation of very long chain fatty acids like 2; *OSBPL8*: oxysterol binding protein like 8.

2 F: forward; R: reverse.

### PCR amplification and DNA sequencing

PCR amplifications were performed in a 20.0 μL reaction volume, which included 10.0 μL 2 × Hieff PCR Master Mix (Yeasen Biotechnology, Shanghai, China), 1.0 μL of upstream and downstream primers, 1.0 μL DNA sample, and 7.0 μL double-distilled water. The reaction conditions were as follows: initial denaturation at 94°C for 10 min; 34 cycles of denaturation at 94°C for 30 s, annealing (Table 1) for 30 s, and extension at 72°C for 30 s, followed by a final extension at 72°C for 10 min. The amplified products were stored at 4°C. The PCR products of *OSBPL8* and *ELOVL2* were sequenced by Beijing Tsingke Biological Co. Ltd. (Beijing, China). Sequence chromatograms were visualized and analyzed using SnapGene Viewer 6.0.2 (GSL Biotech LLC, Chicago, IL, USA), the peak map of the sequencing results was generated, and the genotypes of polymorphic sites were recorded.

### Statistical analysis

The allele frequency, genotype frequency, heterozygosity (**He**), effective allele number (**Ne**), polymorphism information content (**PIC**), Chi-square (**χ^2^**) test, and Hardy‒Weinberg equilibrium *P*-value were calculated using Microsoft Excel 2021 (Microsoft Corp., Redmond, WA, USA) with custom formulas. Loci were classified based on the PIC values: low polymorphism (PIC < 0.25), moderate polymorphism (0.25 < PIC < 0.5), and high polymorphism (PIC > 0.5). Associations between SNP genotypes and FE traits in meat-type ducks were evaluated using a general linear mixed model in SPSS 26.0 (IBM Corp., Armonk, NY, USA) with the following model:

$$Y_{\text{ijk}}=\mu +B_{i}+S_{j}+F_{k}+e_{\text{ijk}}$$

where Y_ijk_ represents the phenotypic measurement of the individual, *μ* denotes the overall population mean; B_i_ indicates the fixed effect of batch, S_j_ corresponds to the fixed effect of SNP or combined genotype; F_k_ represents the random effect of family and e_ijk_ represents the random residual error. Multiple testing correction for all probabilities was performed by false discovery rate. The results are expressed as mean ± standard deviation. Statistical significance was set at *P* < 0.05.

## Results

### Descriptive statistics of growth and feed efficiency traits

At 42 d of age, mean body weight (**BW₄₂**) was 3779.01 ± 284.38 g (Table 2). ADFI showed moderate variation (263.78 ± 25.29 g, CV = 9.59%). ADG was 129.74 ± 11.97 g from 21 to 42 d of age. Metabolic body weight (**MBW^0.75^**) was 344.53 ± 18.05 g (CV = 5.24%). FCR exhibited low variation [mean: 2.04 ± 0.11 g/g (CV = 5.38%), range: 1.73–2.44 g/g], whereas RFI showed the highest phenotypic variation (–0.83 ± 13.06 g/d).

**Table 2:** Descriptive statistics of growth performance and feed efficiency traits of meat-type ducks.

Traits^1^	Mean	Minimum	Maximum	SD	CV (%)
BW_42_ (g)	3779.01	2800.00	4535.00	284.38	7.53
ADFI (g/d)	263.78	180.29	321.05	25.29	9.59
ADG (g/d)	129.74	89.71	160.24	11.97	9.23
MBW^0.75^ (g/d)	344.53	282.10	391.19	18.05	5.24
FCR (g/g)	2.04	1.73	2.44	0.11	5.38
RFI (g/d)	−0.83	−52.82	46.86	13.06	-

1 BW_42_: body weight at 42 d of age; ADFI: average daily feed intake; ADG: average daily gain; MBW^0.75^: metabolic body weight; FCR: feed conversion ratio; RFI: residual feed intake.

### Genetic polymorphisms in *ELOVL2* and *OSBPL8*

**Fig. 1:**
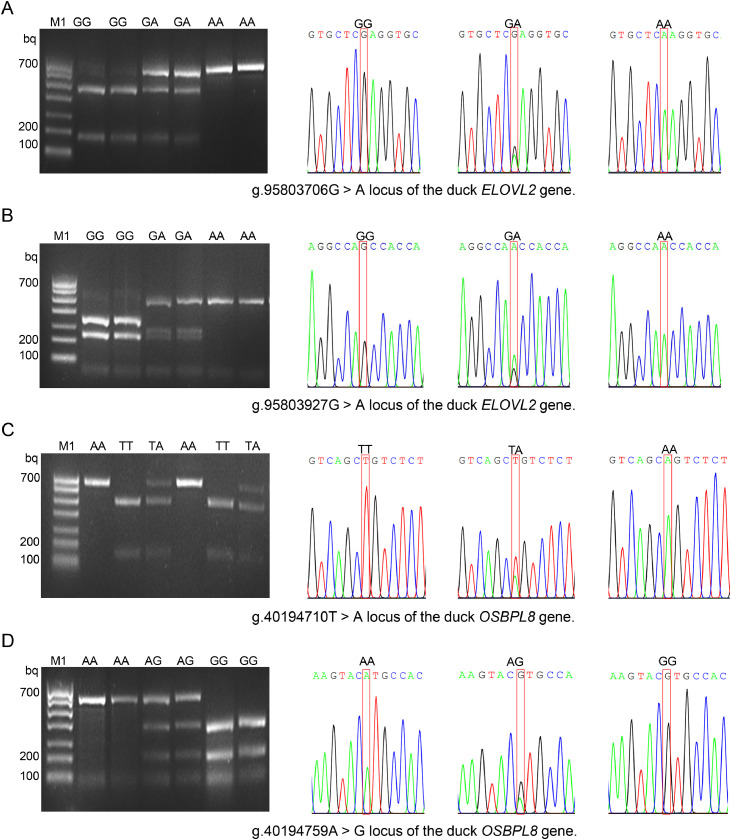
Enzymatic typing and sequencing validation of SNPs of *ELOVL2* and *OSBPL8*. (A) Agarose gel electrophoresis results of the g.95803706 G > A locus of duck *ELOVL2* and the peak maps of the g.95803706 G > A site in *ELOVL2*. (B) Agarose gel electrophoresis results of the g.95803927 G > A locus of duck *ELOVL2* and the peak maps of the g.95803927 G > A site in *ELOVL2*. (C) Agarose gel electrophoresis results of the g.40194710T > A locus of duck *OSBPL8* and the peak maps of the g.40194710T > A site in *OSBPL8*. (D) Agarose gel electrophoresis results of the g.40194759A > G locus of duck *OSBPL8* and the peak maps of the g.40194759A > G site in *OSBPL8.*

Four SNPs were identified in *ELOVL2* and *OSBPL8* (Table 3), including two loci in the promoter region of *ELOVL2*, g.95803706 G > A and g.95803927 G > A, and two loci in *OSBPL8*, g.40194710T > A and g.40194759A > G. All the four loci were in Hardy–Weinberg equilibrium (g.95803706 G > A: χ² = 1.6388, g.95803927 G > A: χ² = 0.1540, g.40194710T > A: χ² = 3.5119, and g.40194759A > G: χ² =2.4857). The PIC values (0.3068, 0.1841, 0.2759, and 0.3720, respectively) indicate low to moderate genetic diversity (Fig. 1).

**Table 3:** Genotypes, allele frequencies, and diversity parameters of polymorphisms in *ELOVL2* and *OSBPL8* of meat-type ducks.

Genes^1^	Endonuclease	Locus	Genotype frequency (%)		Allele frequency (%)		Chi-square test^2^		Genetic polymorphism^3^			
							χ^2^	*P*	Ho	He	Ne	PIC
*ELOVL2*	*PspXI*	g.95803706 G > A	GG (27) GA (202) AA (276)	0.0535 0.4000 0.5465	G (256) A (754)	0.2535 0.7465	1.6388	0.4407	0.6216	0.3784	1.6089	0.3068
	*BstC8I*	g.95803927 G > A	GG (20) GA (154) AA (331)	0.0396 0.3050 0.6554	G (194) A (816)	0.1921 0.8079	0.1540	0.9259	0.7948	0.2052	1.2581	0.1841
*OSBPL8*	*BsaAⅠ*	g.40194710T > A	TT (29) TA (153) AA (323)	0.0574 0.3030 0.6396	T (211) A (799)	0.2089 0.7911	3.5119	0.1727	0.6695	0.3305	1.4937	0.2759
	*PvuⅡ*	g.40194759A > G	AA (164) AG (232) GG (109)	0.3248 0.4594 0.2158	A (560) G (450)	0.5545 0.4455	2.4857	0.2886	0.5059	0.4941	1.9766	0.3720

1 *ELOVL2*: elongation of very long chain fatty acids like 2; *OSBPL8*: oxysterol binding protein like 8.

2 *P*: the χ^2^ test of Hardy−Weinberg equilibrium. A *p*-value > 0.05 indicates that it is in Hardy-Weinberg equilibrium.

3 Ho: observed heterozygosity; He: expected heterozygosity; Ne: effective number of alleles; PIC: polymorphism information content.

### Association between *ELOVL2* polymorphisms and FE traits

Both SNPs in the *ELOVL2* promoter region were significantly associated with FE traits but not with growth-related metrics (Table 4). Specifically, both loci exhibited strong associations with FCR, RFI (both *P* < 0.01), and ADFI (*P* < 0.05), but no associations were observed with BW₄₂, ADG, or MBW^0.75^ (*P* > 0.05).

**Table 4:** Association analysis of *ELOVL2* and *OSBPL8* polymorphisms with growth performance and feed efficiency traits in meat-type ducks.

Traits^1^	*P*-value for significant test			
	*ELOVL2*^2^		*OSBPL8*^3^	
	g.95803706 G > A	g.95803927 G > A	g.40194710T > A	g.40194759A > G
BW_42_ (g)	0.282	0.301	0.449	0.103
ADFI (g/d)	0.011*	0.016*	0.021*	0.146
ADG (g/d)	0.369	0.328	0.429	0.349
MBW^0.75^ (g/d)	0.225	0.299	0.471	0.039*
FCR (g/g)	0.007⁎⁎	0.008⁎⁎	0.017*	0.117
RFI (g/d)	0.001⁎⁎	0.006⁎⁎	0.002⁎⁎	0.017*

1 BW_42_: body weight at 42 d of age; ADFI: average daily feed intake; ADG: average daily gain; MBW^0.75^: metabolic body weight; FCR: feed conversion ratio; RFI: residual feed intake.

2 *ELOVL2*: elongation of very long chain fatty acids like 2.

3 *OSBPL8*: oxysterol binding protein like 8.

⁎ *P* < 0.05,.

⁎⁎ *P* < 0.01.

At the g.95803706 G > A locus, A-allele homozygous (**AA**) and heterozygous (**GA**) individuals had significantly higher ADFI, FCR, and RFI than the G-allele homozygous (**GG**) individuals (*P* < 0.05). No significant differences in BW₄₂, ADG, or MBW^0.75^ were detected among the three genotypes. At the g.95803927 G > A locus, AA and GA individuals had higher ADFI and RFI values than GG birds (*P* < 0.05). GA heterozygotes also showed higher FCR than GG homozygotes (*P* < 0.05). No significant effects were observed for any of the growth traits across the three genotypes (Table 5).

**Table 5:** Least squares mean ± SE for growth performance and feed efficiency traits among SNP genotypes in *ELOVL2* and *OSBPL8*.

Genes^1^	Locus	Genotype	BW^2^_42_ (g)	ADFI^3^ (g/d)	ADG^4^ (g/d)	MBW^0.7^^5^ (g/d) 5	FCR^6^ (g/g)	RFI^7^ (g/d)
*ELOVL2*	g.95803706 G > A	GG (27)	3721.30±359.92	249.93±28.17^b^	126.95±14.59	341.38±23.15	1.97±0.10^B^	−10.09±11.99^B^
		GA (202)	3800.00±287.31	265.50±24.70^a^	130.33±12.24	346.11±18.15	2.04±0.11^A^	−0.60±12.47^A^
		AA (276)	3769.29±273.65	263.87±25.11^a^	129.58±11.48	343.68±17.38	2.04±0.11^A^	−0.10±13.28^A^
	g.95803927 G > A	GG (20)	3684.00±403.18	249.82±30.20^b^	125.83±15.64	338.58±26.00	1.99±0.11^B^	−7.25±12.24^B^
		GA (154)	3788.34±286.79	266.69±25.02^a^	129.99±12.22	345.25±18.03	2.05±0.11^A^	1.43±13.14^A^
		AA (331)	3780.41±274.64	263.27±24.86^a^	129.86±11.59	344.55±17.48	2.03±0.11^AB^	−1.50±12.90^A^
*OSBPL8*	g.40194710T > A	TT (29)	3743.10±294.87	254.12±27.18^b^	127.60±12.11	343.08±18.70	1.99±0.09^b^	−7.94±13.38^B^
		TA (153)	3761.21±277.34	261.35±24.11^ab^	129.20±11.43	343.24±17.81	2.03±0.11^ab^	−2.04±13.11^A^
		AA (323)	3790.67±286.89	265.80±25.44^a^	130.19±12.21	345.27±18.12	2.04±0.11^a^	0.38±12.79^A^
	g.40194759A > G	AA (164)	3770.95±297.08	265.59±25.80	129.72±12.06	343.66±19.18^ab^	2.05±0.11	1.55±13.23^a^
		AG (232)	3804.89±274.82	264.42±24.73	130.39±11.82	346.57±17.22^a^	2.03±0.10	−1.98±12.66^b^
		GG (109)	3736.06±281.51	259.68±25.50	128.37±12.14	341.47±17.63^b^	2.03±0.12	−1.99±13.28^b^

1 *ELOVL2*: elongation of very long chain fatty acids like 2; *OSBPL8*: oxysterol binding protein like 8.

2 BW_42_: body weight at 42 d of age.

3 ADFI: average daily feed intake.

4 ADG: average daily gain.

5 MBW^0.75^: metabolic body weight.

6 FCR: feed conversion ratio.

7 RFI: residual feed intake. ^a-c^ Among the genotypes within each SNP for each trait, different superscript symbols within a column indicate significant differences (*P* < 0.05). ^A-C^ Among the genotypes within each SNP for each trait, different superscript symbols within a column indicate significant differences (*P* < 0.01).

### Association between *OSBPL8* polymorphisms and FE traits

The two SNPs in *OSBPL8* exhibited distinct patterns of association with FE- and growth-related traits (Table 4). The g.40194710T > A locus was significantly associated with the RFI (*P* < 0.01) and ADFI and FCR (both *P* < 0.05), but no associations were detected for BW_42_, ADG, or MBW^0.75^ (*P* > 0.05). In contrast, the g.40194759A > G locus was associated with MBW^0.75^ and RFI (*P* < 0.05) but not with BW_42_, ADFI, ADG, or FCR (*P* > 0.05; Table 4).

At the g.40194710T > A locus, AA homozygotes had significantly higher ADFI and FCR values than the TT homozygotes. Both AA and AT individuals had higher RFI values than TT individuals (*P* < 0.05; Table 5). No significant differences in BW_42_, ADG, or MBW^0.75^ were observed among the three genotypes. At the g.40194759A > G locus, AG individuals had a significantly higher MBW^0.75^ than GG individuals (*P* < 0.05). AA homozygotes exhibited a higher RFI than GG individuals (*P* < 0.05). Notably, no significant differences in BW_42_, ADFI, ADG, or FCR were observed across genotypes (Table 5).

### Association between combined *ELOVL2-OSBPL8* genotypes and FE traits

A total of 42 multi-locus genotype combinations were observed across the four SNPs. To ensure statistical robustness, only the seven most frequent combined genotypes (≥5%) were retained for association analysis (Table 6). No differences were detected among these genotypes for BW₄₂, ADFI, ADG, and MBW^0.75^ (*P* > 0.05).

**Table 6:** Frequency analysis of combined SNP genotypes of *ELOVL2* and *OSBPL8*.

Combined genotype	Number	Frequency (%)	Combined genotype	Number	Frequency (%)
AAAAAAAA	44	8.71	GAGAAAAA	16	3.17
AAAAAAAG	76	15.05	GAGAAAAG	43	8.51
AAAAAAGG	51	10.10	GAGAAAGG	30	5.94
AAAATAAA	38	7.52	GAGATAAA	20	3.96
AAAATAAG	40	7.92	GAGATAAG	20	3.96
AAAATAGG	2	0.40	GAGATAGG	1	0.20
AAAATTAA	10	1.98	GAGATTAA	4	0.79
AAAATTAG	4	0.79	GAGATTAG	4	0.79
AAGAAAAG	4	0.79	GGAAAAGG	1	0.20
AAGAAAGG	1	0.20	GGAATTGG	1	0.20
AAGATAGG	1	0.20	GGGAAAAA	1	0.20
AAGGAAAA	2	0.40	GGGAAAAG	3	0.59
AAGGTAAA	1	0.20	GGGATAAA	3	0.59
AAGGTAAG	2	0.40	GGGATAAG	2	0.40
GAAAAAAA	9	1.78	GAAATTAG	2	0.40
GAAAAAAG	22	4.36	GGGGAAAA	1	0.20
GAAAAAGG	10	1.98	GGGGAAAG	1	0.20
GAAATAAA	9	1.78	GGGGAAGG	8	1.58
GAAATAAG	7	1.39	GGGGTAAA	2	0.40
GAAATAGG	3	0.59	GGGGTAAG	2	0.40
GAAATTAA	2	0.40	GGGGTTAA	1	0.20

Conversely, significant differences were detected in both FCR and RFI. The combined genotype AAAATAAG exhibited the lowest FCR (1.99 ± 0.09 g/g), lower than that of the AAAATAAA genotype (2.07 ± 0.11 g/g, *P* < 0.05). Similarly, the AAAATAAG genotype showed a negative RFI value (–7.11 ± 11.39 g/d), differing significantly from the AAAATAAA genotype (3.68 ± 12.98 g/d, *P* < 0.05). Across all comparisons (Table 7), the AAAATAAG genotype consistently demonstrated lower FCR and RFI values than the other six combined genotypes (*P* < 0.05).

**Table 7:** Least squares mean ± SE for growth performance and feed efficiency traits among combined SNP genotypes in *ELOVL2* and *OSBPL8*.

Combined genotype	BW^1^_42_ (g)	ADFI^2^ (g/d)	ADG^3^ (g/d)	MBW^0.7^^5^ (g/d) 4	FCR^5^ (g/g)	RFI^6^ (g/d)
AAAAAAAA	3787.61±294.62	267.19±27.56	130.31±12.09	344.79±19.30	2.05±0.13^a^	1.89±14.30^a^
AAAAAAAG	3807.50±262.79	267.17±23.48	130.90±10.84	346.29±16.95	2.04±0.10^a^	0.57±12.84^a^
AAAAAAGG	3724.02±249.92	261.21±22.09	128.40±11.24	340.19±15.06	2.04±0.11^a^	0.13±12.14^a^
AAAATAAA	3750.39±240.80	266.28±21.86	128.87±9.93	342.49±15.76	2.07±0.11^a^	3.68±12.98^a^
AAAATAAG	3762.88±255.23	256.26±22.34	128.82±10.48	343.86±16.37	1.99±0.09^b^	−7.11±11.39^b^
GAGAAAAG	3770.93±290.09	265.99±24.10	128.68±12.04	344.85±18.53	2.07±0.10^a^	2.21±11.20^a^
GAGAAAGG	3783.00±258.75	265.22±20.53	129.59±11.79	345.17±15.48	2.05±0.12^a^	0.32±10.97^a^

1 BW_42_: body weight at 42 d of age.

2 ADFI: average daily feed intake.

3 ADG: average daily gain.

4 MBW^0.75^: metabolic body weight.

5 FCR: feed conversion ratio.

6 RFI: residual feed intake. ^a-c^ Among the genotypes within each SNP for each trait, different superscript symbols within a column indicate significant differences (*P* < 0.05).

## Discussion

FE remains a primary target in modern poultry breeding because of its direct impact on production costs and environmental sustainability. Among the available metrics, RFI is increasingly favored over FCR because it reflects metabolic efficiency independent of growth rate (Wen et al., 2018; Ye et al., 2025a). Although interest in the genetic basis of RFI in ducks has grown (Bai et al., 2024; Geng et al., 2025; Guo et al., 2022; Shui et al., 2023), the underlying genetic effects remain poorly understood, highlighting the need for candidate gene validation and functional marker development.

Therefore, this study investigated the potential associations between the candidate genes *OSBPL8* and *ELOVL2* and FE in meat-type ducks. All four loci were suitable for the association analysis because they were in Hardy–Weinberg equilibrium (Ye et al., 2025b). Single-locus analyses revealed significant associations between both genes and FE-related traits. For *OSBPL8*, g.40194710T > A and g.40194759A > G were significantly associated with RFI. Specifically, the T allele at g.40194710T > A was linked to lower RFI, FCR, and ADFI, indicating superior FE without compromising growth (Bai et al., 2022; Cantalapiedra-Hijar et al., 2024). Similarly, at g.40194759A > G, the G-allele was associated with a lower RFI and metabolic body weight, indicating a potential role in modulating maintenance energy requirements. These genetic associations are consistent with the known biological functions of *OSBPL8*, which encodes a lipid transport protein involved in intracellular cholesterol trafficking and lipid droplet (**LD**) homeostasis (Yan et al., 2008). The deletion of *OSBPL8* has been shown to promote LD and triglyceride accumulation (Pu et al., 2023). Additionally, variants in *OSBPL8* have been associated with backfat thickness in pigs (Ma et al., 2019; Tian et al., 2023), underscoring its role in regulating adiposity. Collectively, our findings identified *OSBPL8* as a key regulator of energy partitioning in ducks and support its potential as a target for marker-assisted selection.

*ELOVL2* catalyzes the elongation of very long-chain fatty acids and is expressed in metabolically active tissues (Chen et al., 2020; Gonzalez-Bengtsson et al., 2016). In this study, *ELOVL2* was significantly associated with FE traits in meat-type ducks. The G alleles at the promoter loci g.95803706 G > A and g.95803927 G > A were consistently linked to lower FI and improved FE, whereas the A-allele was associated with lower FE without compromising growth performance. These findings identified G alleles as promising targets for selecting appropriate breeders for long-term enhancement of FE. *ELOVL2* loci are located within the regions that regulate livestock production traits (Cieslak et al., 2013; Kanaka et al., 2021; Liu et al., 2022; Ostrowska et al., 2021). For example, in the promoter region of the caprine *GDF9* gene, three SNPs influence important production traits, including body conformation and milk production, in Damani goats (Ullah et al., 2023). These promoter-region SNPs may be associated with variation in FE-related traits in ducks (Chen et al., 2020). Consistent with this, the previous study has indicated that *ELOVL2* plays a conserved role in energy homeostasis across species and is genetically associated with FE in chickens (Wen et al., 2021). A zebrafish *ELOVL2* mutant showed strong activation of the lipogenesis and lipid uptake pathways (Li et al., 2024). Moreover, studies on humans and mice have established its essential functions in fatty acid and energy metabolism (Li et al., 2022; Medoro et al., 2025; Vicenzi et al., 2025).

As FE is a polygenic trait influenced by multiple loci (Georges et al., 2019; Wu et al., 2025), we evaluated the combined effects of *OSBPL8*-*ELOVL2* genotypes. After excluding low-frequency combinations, seven multi-locus genotypes were retained for association analysis. The AAAATAAG genotype exhibited significantly lower FCR and RFI than all other genotypic combinations, making it the most feed-efficient genotypic combinations in meat-type ducks and supporting its potential application in marker-assisted low-RFI targeted breeding strategies.

Both single-SNP and combined genotype analyses consistently implicated *OSBPL8* and *ELOVL2* in the presentation of FE traits, reinforcing their utility as genetic markers for selecting feed-efficient ducks. Future studies should focus on validating these associations in independent populations and elucidating the underlying molecular mechanisms for informed development of precise breeding strategies that reduce feed costs and promote sustainable meat production.

## Conclusion

This study demonstrated that polymorphisms in *ELOVL2* (g.95803706 G > A and g.95803927 G > A) and *OSBPL8* (g.40194710T > A and g.40194759A > G) are associated with RFI and FCR in meat-type ducks. Individuals carrying the combined AAAATAAG genotype exhibited exceptionally low RFI and FCR, making it a high-priority genotype for marker-assisted selection. These findings establish *ELOVL2* and *OSBPL8* as functional candidate genes for enhancing FE of meat-type ducks, providing practicable resources for selecting appropriate breeders to improve feed utilization and growth performance. Further investigations are necessary to evaluate the associations of *OSBPL8* and *ELOVL2* polymorphisms with feed efficiency traits across various duck populations. Moreover, additional research is essential to explore the functional roles of these genes in regulating growth and feed efficiency of ducks.

## CRediT authorship contribution statement

**Lianzhen Lu:** Writing – review & editing, Writing – original draft, Visualization, Validation, Formal analysis, Data curation, Conceptualization. **Shuang Gu:** Writing – review & editing, Writing – original draft, Formal analysis, Data curation. **Guiru Qiu:** Investigation, Formal analysis, Data curation. **Taikang Zhang:** Formal analysis, Data curation, Validation. **Zihao Tang:** Data curation, Validation, Visualization. **Haoming Chang:** Formal analysis, Data curation, Validation. **Jiafa Wang:** Investigation, Data curation, Validation. **Zhaoyu Geng:** Data curation, Resources, Supervision, Investigation, Conceptualization. **Sihua Jin:** Writing – review & editing, Writing – original draft, Supervision, Resources, Project administration, Funding acquisition, Conceptualization.

## Disclosures

The authors declare that they have no conflict of interest.

## Appendix Supplementary materials

Image, application 1
