# Supplementary material for: Associations between polymorphisms in ELOVL2 and OSBPL8 genes and feed efficiency in meat-type ducks
Source: Poult Sci. 2026 May 13;105(10):107118. doi: 10.1016/j.psj.2026.107118 (PMC13312129; doi:10.1016/j.psj.2026.107118)
Supplement: Supplementary file 1 [file mmc1.pdf]

Editing Certificate

Issued On January 29, 2026

This document certifies that the manuscript listed below has been edited to ensure language and grammar accuracy and is error free in these aspects. The edit was performed by professional editors at Editage, a brand of Cactus Communications. The author's core research ideas were not altered in any way during the editing process. The quality of the edit has been guaranteed, with the assumption that our suggested changes have been accepted and the text has not been further altered without the knowledge of our editors.

Manuscript Title

Associations between polymorphisms in ELOVL2 and OSBPL8 genes and feed efficiency in meat-type ducks

Authors

Lianzhen Lu, Shuang Gu, Guiru Qiu, Taikang Zhang, Zihao Tang, Haoming Chang, Jiafa Wang, Zhaoyu Geng, Sihua Jin*

GAOJI_41_2

Job Code

Prabh Grewal, Senior Vice President, Editage

THE editage PROMISE

QUALITY GUARANTEE • QUALITY GUARANTEE • QUALITY GUARANTEE

VERIFY AT
https://www.editage.cn/ecverify

VERIFICATION CODE
EC-260507-UZ2CBE
